# Synthesis, Structure, Morphology, and Luminescent Properties of Ba_2_MgWO_6_: Eu^3+^ Double Perovskite Obtained by a Novel Co-Precipitation Method

**DOI:** 10.3390/ma13071614

**Published:** 2020-04-01

**Authors:** Thi Hong Quan Vu, Bartosz Bondzior, Dagmara Stefańska, Natalia Miniajluk, Przemysław J. Dereń

**Affiliations:** Institute of Low Temperature and Structure Research, Polish Academy of Science, Okólna 2, 50-422 Wroclaw, Poland; q.vu@intibs.pl (T.H.Q.V.); b.bondzior@intibs.pl (B.B.); d.stefanska@intibs.pl (D.S.); n.miniajluk@intibs.pl (N.M.)

**Keywords:** double perovskite, Eu^3+^, high symmetry site, inversion center, co-precipitation method

## Abstract

Eu^3+^ doped Ba_2_MgWO_6_ (BMW) double-perovskite was successfully synthesized for the first time by the co-precipitation method. The synthesis procedure, crystal structure, as well as morphology of obtained samples are presented. Domination of the ^5^D_0_–^7^F_1_ magnetic–dipole over forced electric–dipole transitions in the emission spectra indicates that Eu^3+^ ions are located in the high symmetry site with inversion center. Only one emission line assigned to the ^5^D_0_–^7^F_0_ transition was observed, confirming that europium substituted for only one host cation site. The photoluminescence excitation (PLE) spectrum is dominated by a strong and broad band related to the O^2−^ → Eu^3+^ and O^2−^ → W^6+^ charge transfer. The decay of the emission from the ^5^D_0_ and ^5^D_1_ levels was investigated. The temperature-dependent emission spectra showed that the T_0.5_ is equal to 350 K. Extinguishing mechanisms of the Eu^3+^ luminescence in the studied host are discussed.

## 1. Introduction 

Recently, Ba_2_MgWO_6_ (BMW), a perfect cubic crystal structure and very promising representative of double perovskite family, has been put into research in the field of ceramic fabrication by virtue of its excellent dielectric properties [1,2]. However, very few studies have discussed its luminescent properties in detail. Indeed, there are only two studies associated with this host doped with Eu^3+^ ions [3,4]. Still, in both cases, the solid-state method was employed to obtain BMW: Eu^3+^.

The chemical formula of a double perovskite is described as A_2_BB’O_6_ in which A^2+^ ions are located in 12-coordination sites while B^2+^ and B’ ions are located in 6-coordination sites [5]. The visualization of the Ba_2_MgWO_6_ crystal structure with all details was published in our previous study [3]. In the BMW structure, Ba^2+^ is located in the site with T_d_ symmetry. As we showed previously, the trivalent europium ions replace the Mg^2+^ in the site with O_h_ symmetry with inversion center [3].

In this study, a series of Ba_2_MgWO_6_: Eu^3+^ was synthesized for the first time by using the co-precipitation method. To exclude defects when the trivalent ion replaces the bivalent one, a Li^+^ co-dopant has been added to locally compensate for the charge. Li^+^ was chosen because of similar ionic radii, which are as follows for ions with six-fold coordination: 108.7, 90, and 86 pm for Eu^3+^, Li^+^, and Mg^2+^, respectively [6]. The charge compensation strategy could be described in this way:

2Mg^2+^ → Eu^3+^ + Li^+^

The purpose of this work is to obtain BMW by wet chemistry methods, i.e., co-precipitation. Of the various synthesis methods, the co-precipitation one is expected to produce material with smaller particle sizes and more homogeneous morphology. A lower sintering temperature contributes to lower energy consumption compared to other synthesis methods [5]. The usefulness of this method will be assessed by comparing the results obtained with the results already published for the solid-state samples. Here, we present the absorbance, excitation, and emission spectra as well as the emission decay profiles. The influence of temperature and dopant concentration on luminescence was also studied.

## 2. Experimental

### 2.1. Synthesis

**Figure 1:**
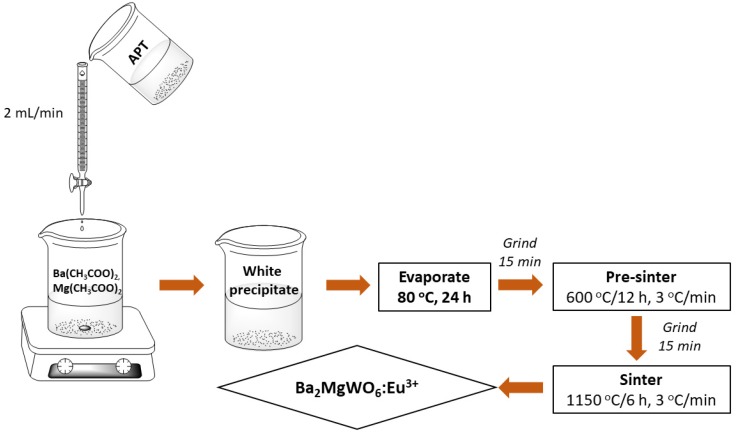
Scheme of Ba_2_MgWO_6_: Eu^3+^ preparation using co-precipitation method.

In this study, concentration series of Ba_2_MgWO_6_: x% Eu^3+^ double perovskite structure (x = 0.1%, 0.5%, 1%, 3%, 4%, 5%, and 7%) were synthesized first time by co-precipitation method, with lower sintering temperature, in comparison with other methods [3,4,7]. To compensate for the evaporation of magnesium ions during the sintering process, a 20% excess of magnesium ions was applied. Ba(CH_3_COO)_2_ (Alfa Aesar, 99%), Mg(CH_3_COO)_2_∙4H_2_O (Alfa Aesar, 99.95%), (NH_4_)_10_H_2_(W_2_O_7_)_6_ (Sigma–Aldrich, 99.99%), Eu(CH_3_COO)_3_ (Alfa Aesar, 99.9%), were used as starting materials. Firstly, the stoichiometric quantities of barium acetate Ba(CH_3_COO)_2_, magnesium acetate Mg(CH_3_COO)_2_∙4H_2_O and ammonium paratungstate (NH_4_)_10_H_2_(W_2_O_7_)_6_ (APT) were dissolved separately in distilled water. Next, a white precipitate was formed immediately after the first droplet of APT solution added slowly (2 ml/min) under stirring 200 rpm, 25 °C. The precipitate was evaporated by heating at 80 °C for 20 h before pre-sintering at 600 °C for 12 h. The final annealing was carried out at 1150 °C for 6 h, with the constant heating rate, 3 °C/min using corundum crucible. After each step, the obtained products were ground for 15 minutes. The scheme of BMW preparation is presented in Figure 1.

### 2.2. Characterization 

The structure of materials was analyzed by X’Pert ProPANalytical X-ray diffractometer (PANalytical, Almelo, The Netherlands) using Cu K_α_ radiation (λ = 1.54056 Ǻ) in a 2θ range from 10° to 90° with a step of 0.026°. For measurements of the reflectance absorption spectra, the Varian Cary 5E UV–Vis-NIR spectrophotometer (Agilent, Santa Clara, CA, USA) was used. The emission spectra in the temperature range 10–300 K were recorded using a Jobin-Yvon monochromator (Horiba Scientific, Kyoto, Japan), along with a closed-cycle helium cryostat. Decay profiles were recorded with a Lecroy digital oscilloscope (Teledyne LeCroy, New York, NY, USA) with the Nd: YAG laser as the excitation source. Scanning electron microscope FEI NOVA NanoSEM230 (FEI, Hillsboro, OR, USA) was used to characterize the morphology of sample. The excitation spectrum was recorded using a McPherson spectrometer with a 150 W xenon lamp as the excitation source and a Hamamatsu R928 photomultiplier (Hamamatsu Photonics K.K, Shizuoka, Japan) as the detector. Temperature-dependent emission spectra were measured with the Hamamatsu Photonic multichannel analyzer PMA-12 equipped with a BT-CCD linear image sensor (Hamamatsu Photonics K.K, Shizuoka, Japan). The temperature of the samples during emission measurements was controlled by the Linkam THMS 600 Heating/Freezing Stage (The McCRONE group, Westmont, IL USA).

## 3. Results and Discussion

**Figure 2:**
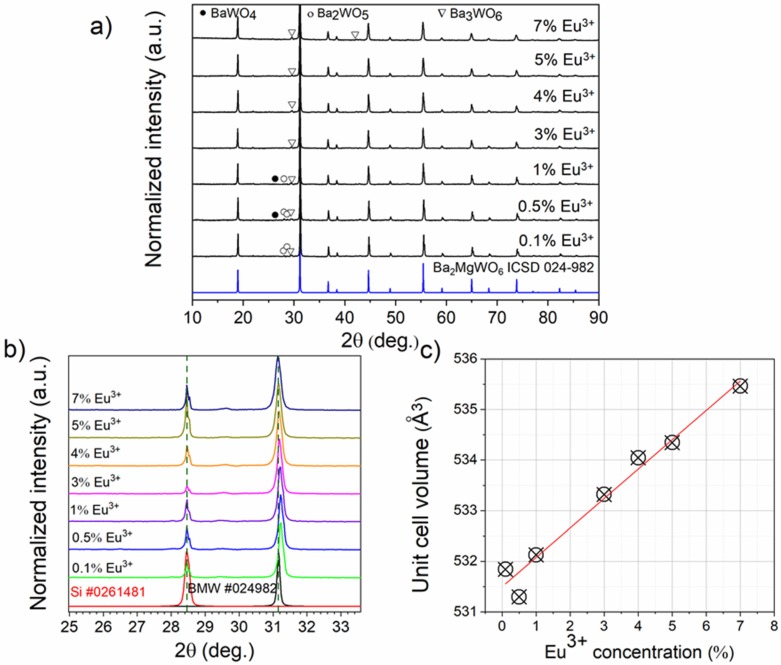
(**a**) The X-ray powder diffraction of Ba_2_MgWO_6_: x% Eu^3+^, (x = 0.1, 0.5, 1, 3, 4, 5, 7 %) (**b**) the shifting of position of diffraction lines with Eu^3+^ concentration and (**c**) The changing of unit cell volume as a function of Eu^3+^ concentration.

Ba_2_MgWO_6_ possesses the cubic crystal structure with a rock salt lattice of which corner-shared MgO_6_ and WO_6_ octahedrons. The Ba cations are located in the 12-fold coordination sites. The crystal structure of BMW was described in detail in our previous study [3]. The X-ray diffractograms of Ba_2_MgWO_6_: x Eu^3+^ samples (x = 0.1%, 0.5%, 1%, 3%, 4%, 5%, and 7%) are shown in Figure 2. The X-ray diffraction patterns of prepared samples match well with the Ba_2_MgWO_6_ pattern (ICSD card number 024-982) with the space group Fm-3m. The higher concentration of europium causes the additional diffraction peaks to appear. The alien phases belong to the barium tungstate family, including BaWO_4_ (present at 2θ of 26.4°), Ba_2_WO_5_ (28°, 28.6°, 29.6°), and Ba_3_WO_6_ (29.4°, 42°) these phases were also mainly found in other reports [1,7]. Fortunately, the alien phases did not affect the luminescence properties as well as further ceramic preparation. The positions of the XRD peaks were corrected with the aid of Si admixture (ICSD card number 026-1481). Ba^2+^ is a very large ion, its crystal radius (CR) for coordination number equal 12 (CN = 12) is 175 pm. While the CR of the Eu^3+^ ion for CN = 9 is-Shannon gives no data for the highest CN-only 126 pm [6]. If europium ions have replaced barium ions, then the unit cell of BMW should shrink. Since it is growing (see Figure 2c), we conclude that larger Eu^3+^ ions (CR = 108.7 pm) replace smaller Mg^2+^ ions (CR = 86 pm) in the sites with CN = 6. The replacement of W^6+^ with Eu^3+^ was excluded because of the large difference between the charge of these ions and their crystal radius equal to 74 and 108.7 pm for W^6+^ and Eu^3+^, respectively.

**Figure 3:**
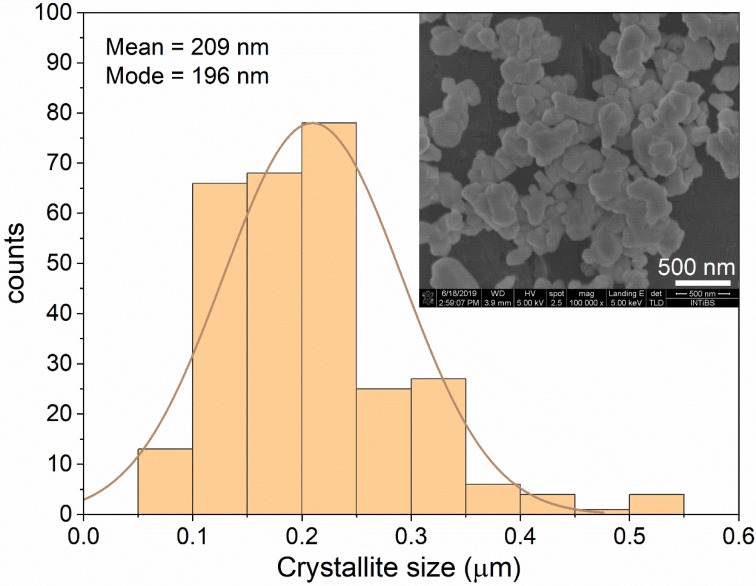
Crystallite size distribution and SEM image of Ba_2_MgWO_6_: 3% Eu^3+^.

The microstructure of the representative sample BMW: 3% Eu^3+^, and the crystallite size distribution of the obtained sample (calculated using ImageJ software) are shown in Figure 3. In general, single grains were not observed in the SEM image. As can be seen, the morphology of the sample was heterogeneous. However, a large number of crystallites were agglomerated, forming large objects with irregular shapes. As a result, the distribution of the crystallite sizes is in a wide range from 70 nm up to 500 nm. The average crystallite size was estimated to be around 209 nm. This value is much smaller than those obtained using a conventional solid-state technique [3,7], demonstrating one of the advantages of the wet chemistry method. This result encourages further work to eliminate agglomeration to ease ceramic preparation.

**Figure 4:**
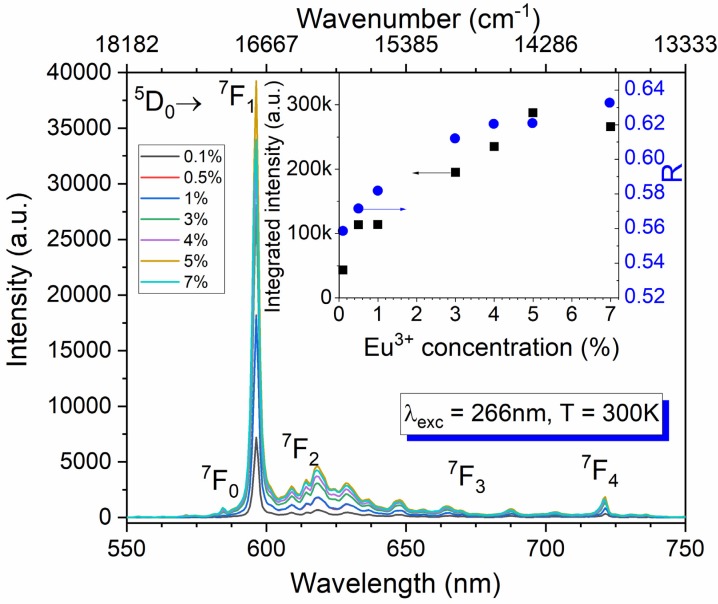
The 300 K emission spectra of Ba_2_MgWO_6_: x% Eu^3+^, (x = 0.1, 0.5, 1, 3, 4, 5, 7%) recorded under 266 nm excitation Nd: YAG line. Inset: the integrated emission intensity and the R parameter (see text for explanation) in function of Eu^3+^ concentration.

The photoluminescence of Ba_2_MgWO_6_ doped with a series of europium concentration from 0.1% to 7% was measured under 266 nm excitation wavelength at room temperature (see Figure 4). The picture is uncommon to majority cases when Eu^3+^ ions are located at the sites without the center of inversion. Here the ^5^D_0_ → ^7^F_1_ magnetic dipole transition dominates the spectrum as sharp and intense peak at 596 nm. The forced electric dipole transitions are much weaker and broadened due to vibronic coupling, assigned to the ^5^D_0_ → ^7^F_J_ transitions (where J = 0, 2, 3, 4) are observed at about 584.4, 618.7, 665, and 721.3 nm, respectively. The integrated emission intensity in the function of the Eu^3+^ concentration was depicted in the inset of Figure 4. The luminescent intensity increases significantly with the increase of Eu^3+^ concentration up to 5%. Above that point, the emission intensity decreases as a consequence of the concentration quenching process.

Due to the hypersensitive nature, the ^5^D_0_ → ^7^F_2_ electric-dipole transition it susceptible to the even small changes of the crystal field. In contrast, the ^5^D_0_–^7^F_1_ magnetic dipole transition is virtually unaffected by neither symmetry site nor host lattice-type. Hence, the asymmetry factor R, the ratio between the integrated intensity of the ^5^D_0_ → ^7^F_2_ and that of ^5^D_0_ → ^7^F_1_ transitions, is often used to determine the changes in the nearest environment of Eu^3+^ in the lattice. Thus, the higher the value R is, the lower the symmetry of the Eu^3+^ ions occupancy site. For the investigated samples, the R parameter increases gradually from 0.56 to 0.63 when dopant concentration increases. It demonstrates a slight distortion of the structure, which insignificantly decreases local symmetry of the Eu^3+^ ions.

**Figure 5:**
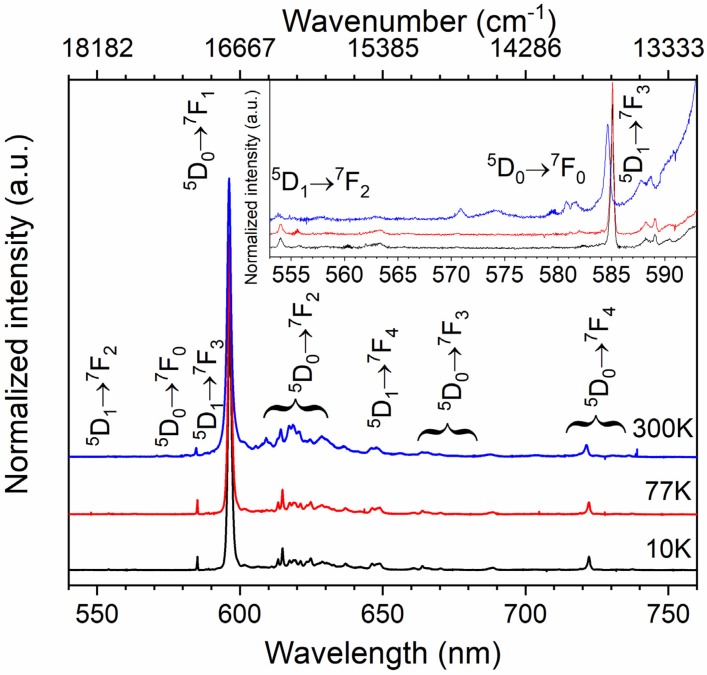
The 300 K (blue), 77 K (red) and 10 K (black) emission spectra of Ba_2_MgWO_6_: 5% Eu^3+^ excited at 266 nm.

To assign every single emission peak, and to determine the number of the Eu^3+^ sites, the emission spectra were recorded at low temperature (77 K and 10 K). The intensity of the f-f lines significantly increased, while the vibronic ones almost disappeared when the temperature decreased down to 10 K (see Figure 5). Only one narrow line at 585 nm attributed to the ^5^D_0_ → ^7^F_0_ transition was observed at room and low (10 and 77 K) temperature. It is apparent evidence of the europium location in the only one crystallographic site (see the inset in Figure 5). Emission spectra were carefully studied. All detected lines were tentatively assigned either to MD or ED or vibronic transitions. Their energies are presented in Supplementary Table S1.

**Figure 6:**
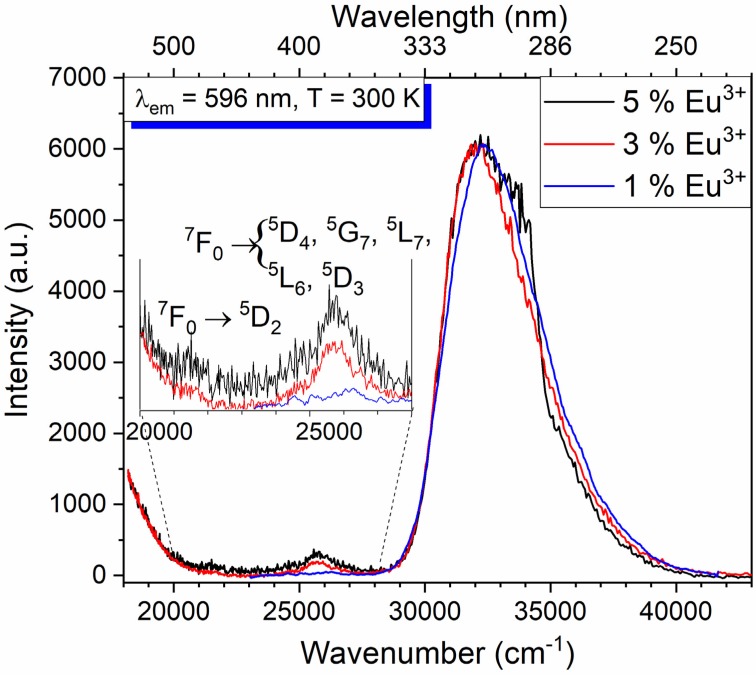
The 300 K excitation spectra of Ba_2_MgWO_6_: 5% Eu^3+^ (black), 3% Eu^3+^ (red) and 1% Eu^3+^ (blue) monitored at the ^5^D_0_–^7^F_1_ transition.

The excitation spectra of the 1%, 3%, and 5% Eu^3+^ doped Ba_2_MgWO_6_ was monitored at 596 nm, i.e.at the maximum of the ^5^D_0_ → ^7^F_1_ transition at room temperature (see Figure 6). The spectra consist of a broad and intense band ranging from 250 nm to 350 nm. The broad band results from two charge transfer transitions (CTB), including the O^2^^−^ → W^6+^ and O^2^^−^ → Eu^3+^ transitions. There is also a small band at about 395 nm assigned to intra-configurational f-f transitions of Eu^3+^ ions, weak due to the Laporte selection rule [8]. They become partially allowed because of the lowering of the symmetry site when introducing a higher amount of Eu^3+^ and Li^+^ ions. Our recent investigation does not reveal any trace of f-f transitions in the excitation spectra, but there effective doping was several times smaller [3]. The sample with 1% Eu^3+^ virtually does not show the f-f transitions. Mainly CTB is observed similarly as in the solid-state method [3]. 

It is not easy to find the energy of the O^2−^ → Eu^3+^ transitions since the amount of dopant is small comparing to the tungstate, which forms the host structure. Analogously, the broad band at 318 nm results from both charge transfer transitions in Ba_3_WO_6_: Eu^3+^ [9]. The energy of the O^2−^ → Eu^3+^ CTB is influenced not only by the ligand electronegativity but also by the local surroundings of the Eu^3+^ ions [8]. The location of the O^2−^–W^6+^ CTB has been reported around 300 nm in g-C_3_N_4_/Ba_2_MgWO_6_: Eu^3+^ [4], 301 nm in Ba_2_MgWO_6_: Eu^3+^ [3], 310 nm in host Ba_2_MgWO_6_ [7], 325 nm [10] in NaLaMgWO_6_: Eu^3+^. As noted in the preliminary work, the O^2−^–Eu^3+^ CTB was found at around 304 nm in CaMoO_4_: Eu^3+^, 293 nm in AMoO_4_: Eu^3+^ (A = Ba, Sr), and at 250 nm in BaNa_2_W_2_O_11_: Eu^3+^. The less intense and narrower bands appeared near the visible region (from 370 to 400 nm) and at 462.5 nm that could be ascribed to f-f transitions of the ^7^F_0_ → ^5^D_4_, ^5^G_7_, ^5^L_7_, ^5^L_6_, ^5^D_3_ and ^7^F_0_ → ^5^D_2_ transitions, respectively, [11,12,13] (see inset in Figure 6). 

**Figure 7:**
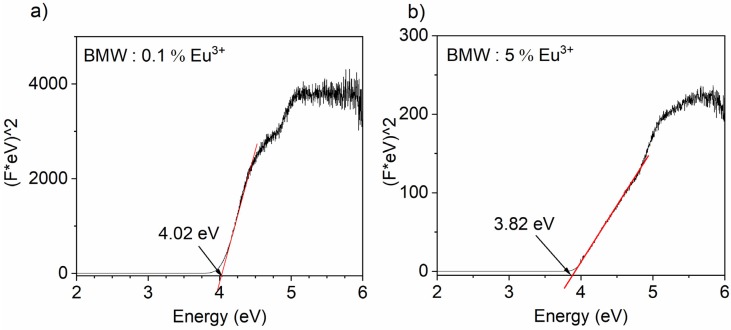
Forbidden energy band-gaps (E_g_) of Ba_2_MgWO_6_: x Eu^3+^, (**a**) x = 0.1%; (**b**) x = 5%) calculated from the absorption spectra.

The absorption spectra registered at 300 K were useful to calculate the energy of the forbidden band-gap of the investigated samples. The band-gap energy E_g_ was determined by applying the modified Kubelka–Munk function and plotted as (F(R).h*v*)^2^ versus h*v*, also called a Tauc method [14]. It was found that the higher dopant content was used, the smaller band-gap energy was obtained, (see Figure 7). For the lowest Eu^3+^ concentration, the band-gap energy was 4.02 eV while for the sample doped with 5% of Eu^3+^, E_g_ = 3.82 eV. 

**Figure 8:**
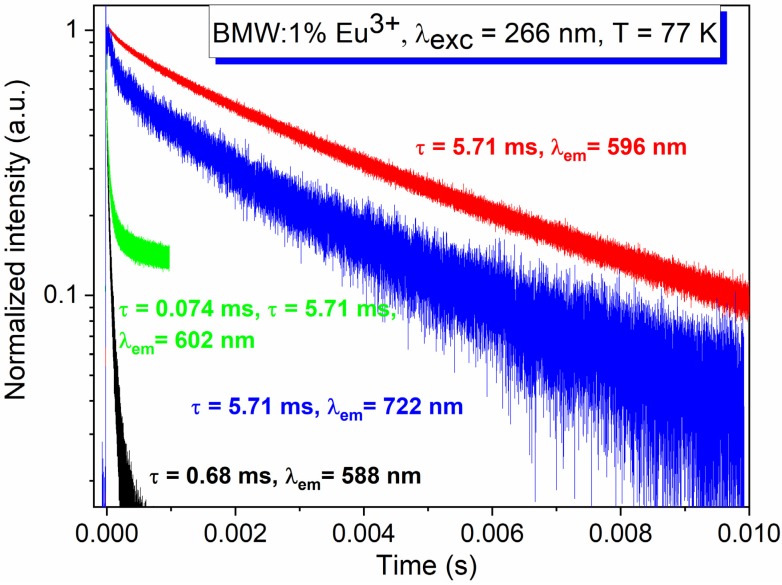
The 77 K decay profiles of Ba_2_MgWO_6_: 1% Eu^3+^ under 266 nm excitation and monitored at different wavelength.

The 77 K decay curves of the BMW sample doped with 1% Eu^3+^ was excited at 266 nm and monitored at the different wavelengths corresponding to the transitions from the ^5^D_1_ (588 nm, 602 nm) and the ^5^D_0_ (596 nm, 722 nm) levels (see Figure 8). The decay curves are multi-exponential in all cases. The first component was usually connected with the nonradiative process, while the longer ones resulted from the emissions from different Eu^3+^ energy levels depending on the monitored wavelength. The decay of magnetic-dipole transition consisted of two components, one with the short lifetime-related to the nonradiative process-and the longer one, τ = 5.71 ms, characteristic for the Eu^3+^ located in the site with very high symmetry. The decay time of the ^5^D_1_ level was found to be equal to 70 μs, it is much smaller than expected, probably because of the fast nonradiative transition to the lower ^5^D_0_ level and also possible cross-relaxation mechanism—the latter will be discussed further.

**Figure 9:**
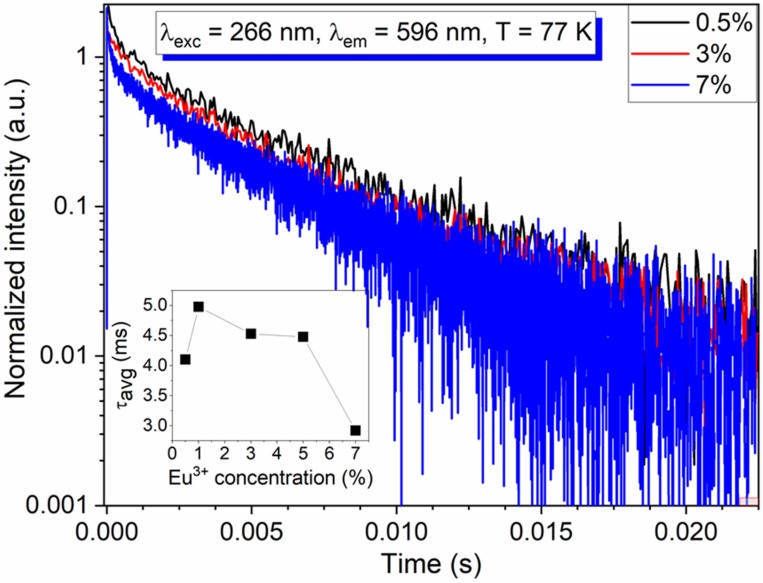
The 77 K decay profiles of Ba_2_MgWO_6_: x Eu^3+^, (x = 0.5, 3, 5, 7%). Inset: the average decay time in function of Eu^3+^ concentration.

The luminescence kinetics were recorded at 77 K upon excitation at 266 nm. Figure 9 presents the ^5^D_0_ → ^7^F_1_ emission decay profiles of the BMW doped with 0.5%, 3%, and 7% of Eu^3+^ ions. The emission decay curves exhibited the characteristics of the nonexponential function. The average decay time can be expressed as follow:

(1) $$\tau_{avg}=\frac{\int_{0}^{\infty }\left(I\left(t\right)\times t\right)dt}{\int_{0}^{\infty }I\left(t\right)dt}≅\frac{\int_{0}^{t^{max}}\left(I\left(t\right)\times t\right)dt}{\int_{0}^{t^{max}}I\left(t\right)dt}$$

where I(t) is the emission intensity at time t, 0 < t < t^max^. The emission average decay time gradually decreased from 5 ms to 2.9 ms with an increase of Eu^3+^ concentration from 1% to 7%, respectively. Two factors could explain this observation, one is the nonradiative processes, which are undoubtedly present. Second the distortion of the Eu^3+^ coordination polyhedra due to the increase of the dopant concentration. We recall here that the effective doping is twice as important as in the previous study [3,4] because of the presence of Li^+^. The distortion causes a slight decrease in the symmetry, so the transition probability increases, although the emission still dominates the MD transition. 

The Judd–Ofelt (J-O) intensity parameters (Ω_λ_, where λ = 2, 4, 6) were calculated on the basis of the BMW: Eu^3+^ (5%) emission spectrum excited at 266 nm and recorded at 300 K. Exactly the same formalism and the same set of equations were used as in our previous work [3]. The refractive index “n” of the BMW host is 1.874 [3]. The matrix elements U(2) = 0.0035, U(4) = 0.003 and U(6) = 0.0005 from the work of K. Binnemans [8] were applied for the calculations.

Using the Ω_λ_ parameters, the A_ij_ transition rates, the β_ij_ branching ratios, were calculated for the ^5^D_0_ → ^7^F_J_ transitions (where J = 1, 2, 4, 6) (see Table 1 and Table 2). A recent analysis in the frame of J-O theory for the BMW: Eu^3+^ (2%) solid-state sample was carried out at 395 nm of excitation [3]. However, there is a possibility that excitation at 395 nm will be absorbed by all Eu^3+^ ions-including those which could be in the additional, foreign phases present in the sample. Consequently, this time, the emission was excited with the 4^th^ harmonic of the Nd: YAG laser reaching the O^2-^ → Eu^3+^ charge transfer band, both for the solid-state and co-precipitation samples.

There is very little difference among J-O parameters between sample synthesized by co-precipitation and solid-state method (see Table 1, Table 2 and Table 3). The value of Ω_2_ is twofold smaller than that of Ω_6_. The increase in the concentration of Eu^3+^ from 2% to 5% leads to a modification of the Ω_λ_ (see Table 3). These are phenomenological parameters which only depend on the matrix. An increase in the concentration of dopant ions having a larger diameter and a different charge than the replaced Mg^2+^ must cause the deformation of the matrix. We must not forget either that with 5% Eu^3+^, we introduce 5% Li^+^. 

The Ω_𝜆_ values found in this work are comparable with other results obtained in similar tungstate double perovskites but with lower symmetry (Li, Na, K)LaMgWO_6_ [15]_._ Due to the fact that Ω_2_ is very sensitive to the angular changes while Ω_4_ and Ω_6_ are mostly affected by bond covalence between dopants and ligands [16]. As a result, a distinction among various tungstate double perovskites was observed, Ω_2_ values of (Li, Na, K)LaMgWO_6_ with lower site symmetry are seven times higher in comparison with those of the higher site symmetry (O_h_) in BMW. The Ω_4_ value of (Li, Na, K)LaMgWO_6_ is also fivefold higher than those of BMW while the Ω_6_ value is quite similar, except for KLaMgWO_6_. 

Besides, the rates of total, radiative, non-radiative transition along with the theoretical radiative and experimental decay time as well as the quantum efficiency of sample BMW: Eu^3+^ are presented in Table 2. For sample prepared by co-precipitation and solid-state, the calculated radiative decay times are 4.96 and 5.31 ms and the quantum efficiencies are 90.2% and 85 %, respectively.

**Figure 10:**
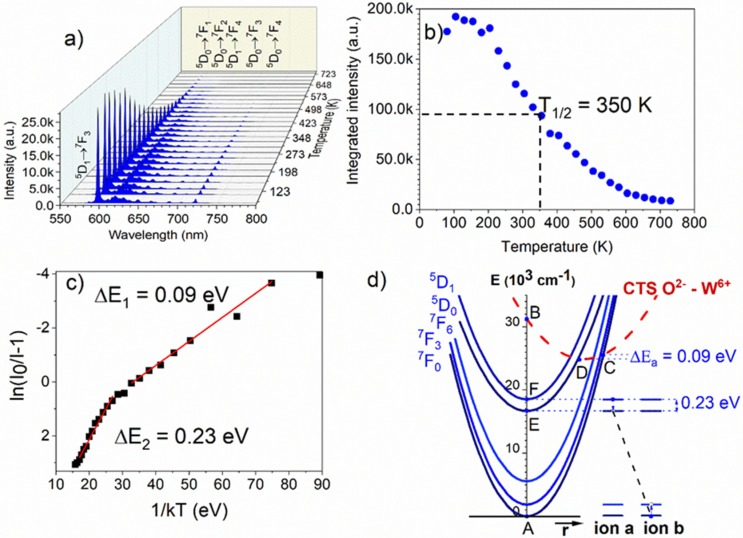
(**a**) The emission spectra of Ba_2_MgWO_6_: 5% Eu^3+^ measured as a function of temperature; (**b**) The integrated intensity as a function of temperature; (**c**) Activated energies (∆E_a_) of two processes calculated from a function of ln(I_0_/I-1) versus 1/kT; (**d**) Simplified single configurational coordinate energy diagram of Ba_2_MgWO_6_: Eu^3+^.

The temperature-dependent emission spectra under excitation at 266 nm were performed in the temperature range from 80–730 K (Figure 10a). The emission intensity does not change much up to 200 K. Above this temperature, emission intensity significantly decreases. The temperature quenching, at which the integrated intensity of emission decreased half in comparison with the initial emission intensity (T_0.5_) was at around 350 K (Figure 10b). The emission intensity of transitions from the ^5^D_1_ level was also quenched, along with the transitions from the ^5^D_0_ level. Figure 10 shows the relationship of ln(I_0_/I-1) versus 1/kT, two mechanisms responsible for emission quenching took place. The activation energies for thermal quenching were calculated to be 0.09 eV and 0.23 eV. The first one is due to the process which operates in the 80–350 K temperature range. Above 350 K, the second process is switched on and is more effective in the emission quenching. The first process is of the same nature as that observed in LaAlO_3_: Eu^3+^ and explained in detail by Blasse [17], but the CT state involved here is associated with the O^2−^ ↔ W^6+^ transition. Here, the same approach was applied to support the single configurational coordinate model depicted in Figure 10d. Upon excitation into the charge transfer state-transition A → B, some part of the excitation energy relaxes to point C and then to Eu^3+^, and the rest, with thermal excitation, flows from the CTS to the Eu^3+^ ground state via point D. (see Figure 10d). The other nonradiative process is switched on above 350 K when the ^5^D_1_ level becomes thermally populated and is due to the following cross-relaxation: [^5^D_1_, ^7^F_0_] → [^5^D_0_, ^7^F_3_] (see points F and E in Figure 10d). Its activation energy (∆E_a_ = 0.23 eV = 1855 cm^−1^) is related to the difference energy between the ^5^D_0_ and ^5^D_1_ and ^7^F_0_–^7^F_3_ levels.

## 4. Conclusions

The co-precipitation method was successfully employed in the synthesis of Ba_2_MgWO_6_ double perovskite doped with Eu^3+^ ions. Dopant replaces with Mg^2+^ and is located at the O_h_ site with the inversion center. The mean size of the particles determined from SEM images was around 209 nm. The single line of the ^5^D_0_–^7^F_0_ transition and the dominance of the ^5^D_0_–^7^F_1_ one are undeniable evidence that europium ions occupy one highly symmetrical site in the BMW host. The strongest emission was observed for 5% of Eu^3+^. The broad band from 250 to 350 nm in the PLE spectrum corresponds to the O^2−^ → Eu^3+^ and O^2−^ → W^6+^ charge transfer transitions. Our results are in good agreement with Blasse’s theory, which describes the relationship between Eu^3+^–O^2−^ distance with localization of CTS maximum and emission efficiency. The thermal quenching investigation showed that cross-relaxation processes generally quench the emission of Eu^3+^ at temperatures higher than 350 K. At lower temperatures, the excitation energy is lost through the crossing point of the CTS state and the Eu^3+^ ground level. We believe that this material will be perfect for producing transparent or translucent ceramics.

## Figures and Tables

**Table 1:** The transition rates, branching ratios for ^5^D_0_ → ^7^F_J_ (J = 1, 2, 4, 6) transitions of BMW: Eu^3+^.

Sample		^5^D_0_ → ^7^F_1_	^5^D_0_ → ^7^F_2_	^5^D_0_ → ^7^F_4_	^5^D_0_ → ^7^F_6_
Co-precipitation	A_0-J_ (s^−1^)	96.35	77.64	17.82	9.61
	Β_0-J_ (%)	47.8	38.55	8.85	4.8
Solid-state	A_0-J_ (s^−1^)	96.35	70.33	14.42	7.13
	Β_0-J_ (%)	51.2	37.4	7.6	3.8

**Table 2:** Total, radiative and non-radiative transition rates, calculated and experimental decay time and quantum efficiency of BMW: Eu^3+^.

	A_tot_ (s^−1^)	A_rad_ (s^−1^)	A_nrad_ (s^−1^)	τ_rad_ (ms)	τ_exp_ (ms)	η (%)
Co-precipitation	223.2	201.4	21.8	4.96	4.48	90.2%
Solid-state	222.2	188.2	34	5.31	4.5	85

**Table 3:** The Judd–Ofelt ***Ω***_𝜆_ ($\times 10^{-20}{\mathrm{cm}}^{2}$) parameters for Eu^3+^ ions in various double perovskites.

	***Ω***_2_	***Ω***_4_	***Ω***_6_	Reference
Ba_2_MgWO_6_: 5% Eu^3+^ (co-precipitation)	1.18	0.48	2.5	This work
Ba_2_MgWO_6_: 2% Eu^3+^ (solid-state)	1.07	0.39	1.83	This work
LiLaMgWO_6_: 0.01% Eu^3+^ (solid-state)	7.27	2.59	1.64	[15]
NaLaMgWO_6_: 0.01% Eu^3+^ (solid-state)	7.29	1.86	1.8	[15]
KLaMgWO_6_: 0.01% Eu^3+^ (solid-state)	7.53	5.32	5.09	[15]

## Supplementary Materials

The following are available online at https://www.mdpi.com/1996-1944/13/7/1614/s1, Table S1. Tentative assignment of the lines from the emission spectra of Ba_2_MgWO_6_: 5% Eu^3+^ based on measurements at 300 K, 77 K and 10 K.
