# Supplementary material for: Synthesis, Structure, Morphology, and Luminescent Properties of Ba2MgWO6: Eu3+ Double Perovskite Obtained by a Novel Co-Precipitation Method
Source: Materials (Basel). 2020 Apr 1;13(7):1614. doi: 10.3390/ma13071614 (PMC7178313; doi:10.3390/ma13071614)
Supplement: Supplementary file 1 [file materials-13-01614-s001.pdf]

materials

Supplementary Materials

Synthesis, Structure, Morphology, and Luminescent Properties of Ba_2MgWO_6: Eu^{3+} Double Perovskite Obtained by a Novel Co-Precipitation Method

Thi Hong Quan Vu, Bartosz Bondzior, Dagmara Stefańska, Natalia Miniajluk and Przemysław J. Dereń *

Institute of Low Temperature and Structure Research, Polish Academy of Science, Okólna2, 50-422 Wroclaw, Poland; q.vu@intibs.pl (T.H.Q.V.); b.bondzior@intibs.pl (B.B.); d.stefanska@intibs.pl (D.S.); n.miniajluk@intibs.pl (N.M.)

* Correspondence: p.deren@intibs.pl

Received: 2 March 2020; Accepted: 26 March 2020; Published: date

Table S1. Tentative assignment of the transitions from the emission spectra of Ba_2MgWO_6: 5%Eu^{3+} at 300 K, 77 K and 10 K.

Wavelength (nm)	Wavenumber (cm^{-1})	Assignment
548.25	18240	$^5D_1 - {}^7F_2 + 2 \times T'$
554	18051	$^5D_1 - {}^7F_2$
554.78	18025	$^5D_0 - {}^7F_0 + T' + \nu_s$
555.76	17993	$^5D_1 - {}^7F_2$
558.5	17905	$^5D_0 - {}^7F_0 + \nu_s$
563.35	17751	$^5D_0 - {}^7F_0 + T' + \nu_{as}$
565.6	17680	$^5D_1 - {}^7F_2 + 3 \times T'$
566.6	17649	$^5D_1 - {}^7F_3 + T' + \nu_{as}$
570.555	17527	$^5D_0 - {}^7F_0 + \delta$
574.1	17419	$^5D_0 - {}^7F_1 + T' + \nu_{as}$
579.4	17259	$^5D_1 - {}^7F_3 + 2 \times T'$
581.1	17209	$^5D_0 - {}^7F_1 + \delta$
582.2	17176	$^5D_1 - {}^7F_2 + 2 \times \delta$
584.1	17120	$^5D_1 - {}^7F_3 + T'$
585	17094	$^5D_0 - {}^7F_0$
588.2	17001	$^5D_1 - {}^7F_3$
589	16978	$^5D_1 - {}^7F_3$
590.4	16938	$^5D_1 - {}^7F_3$
592.5	16878	$^5D_1 - {}^7F_3$
596.3	16770	$^5D_0 - {}^7F_1$
596.5	16764	$^5D_0 - {}^7F_1$
596.8	16756	$^5D_0 - {}^7F_1$
601.6	16622	$^5D_1 - {}^7F_3 + 2 \times T'$
606.5	16488	$^5D_1 - {}^7F_3 + \delta$
608.25	16441	$^5D_0 - {}^7F_2 + 2 \times T'$
609.2	16415	$^5D_0 - {}^7F_2 + 2 \times T'$
611.1	16364	$^5D_1 - {}^7F_3 + T' + \delta$
613.4	16303	$^5D_0 - {}^7F_2$
614.9	16263	$^5D_0 - {}^7F_2$
617.4	16197	$^5D_0 - {}^7F_2$
618.7	16163	$^5D_0 - {}^7F_2$
619.4	16145	$^5D_0 - {}^7F_2 + T'$

621.2	16098	$^5D_0 - {}^7F_1 + T' + \nu_{as}$
623.4	16041	$^5D_0 - {}^7F_2 + T'$
624.8	16005	$^5D_0 - {}^7F_2 + 2 \times T'$
627.5	15936	$^5D_0 - {}^7F_2 + 2 \times T'$
628.8	15903	$^5D_0 - {}^7F_2 + 2 \times T'$
630.2	15868	$^5D_0 - {}^7F_2 + \delta$
631.2	15843	$^5D_0 - {}^7F_1 + 3 \times T' + \nu_{as}$
632.8	15803	$^5D_0 - {}^7F_3 + 2 \times T' + \nu_{as}$
637	15699	$^5D_0 - {}^7F_3 + T' + \delta$
639	15649	$^5D_1 - {}^7F_4$
641.4	15591	$^5D_0 - {}^7F_3 + T' + \delta$
642.5	15564	$^5D_0 - {}^7F_3 + \delta$
646.3	15473	$^5D_0 - {}^7F_3 + T' + \nu_{as}$
648.25	15426	$^5D_0 - {}^7F_2 + 2 \times \delta$
648.9	15411	$^5D_0 - {}^7F_2 + 2 \times T' + \nu_{as}$
651.8	15342	$^5D_0 - {}^7F_3 + \nu_{as}$
656	15244	$^5D_0 - {}^7F_3 + \delta$
661	15129	$^5D_0 - {}^7F_3$
663.9	15063	$^5D_0 - {}^7F_3$
666	15015	$^5D_0 - {}^7F_3$
670.3	14919	$^5D_0 - {}^7F_3$
675.5	14804	$^5D_0 - {}^7F_3$
681.75	14668	$^5D_0 - {}^7F_3 + 2 \times T'$
688.5	14524	$^5D_0 - {}^7F_3 + \nu_{as}$
696.4	14360	$^5D_0 - {}^7F_3 + T' + \nu_{as}$
699.5	14296	$^5D_0 - {}^7F_4 + 2 \times T'$
702.9	14227	$^5D_0 - {}^7F_3 + 2 \times T' + \nu_{as}$
703.4	14217	$^5D_0 - {}^7F_3 + 2 \times T' + \nu_{as}$
708.5	14114	$^5D_0 - {}^7F_3 + 2 \times T' + \delta$
711.8	14049	$^5D_0 - {}^7F_4$
719.2	13904	$^5D_0 - {}^7F_4$
722.2	13847	$^5D_0 - {}^7F_4$
724.8	13797	$^5D_0 - {}^7F_4$
726	13774	$^5D_0 - {}^7F_4$
730	13699	$^5D_0 - {}^7F_3 + 3 \times \delta$
731.9	13663	$^5D_0 - {}^7F_4 + T'$
737.3	13563	$^5D_0 - {}^7F_3 + \nu_s + \nu_{as}$
738.9	13534	$^5D_0 - {}^7F_4 + 3 \times T'$
744	13441	$^5D_0 - {}^7F_3 + 2 \times \nu_s$
751	13316	$^5D_0 - {}^7F_4 + \nu_{as}$
754.8	13249	$^5D_0 - {}^7F_3 + 2 \times \nu_{as} + \nu_s$
760.5	13149	$^5D_0 - {}^7F_4 + 2 \times T' + \delta$

© 2020 by the authors. Submitted for possible open access publication under the terms and conditions of the Creative Commons Attribution (CC BY) license (http://creativecommons.org/licenses/by/4.0/).
